# Measuring the prevalence of sleep disturbances in people with dementia living in care homes: a systematic review and meta-analysis

**DOI:** 10.1093/sleep/zsz251

**Published:** 2019-10-21

**Authors:** Lucy Webster, Sergi Costafreda Gonzalez, Aisling Stringer, Amy Lineham, Jessica Budgett, Simon Kyle, Julie Barber, Gill Livingston

**Affiliations:** 1 Division of Psychiatry, University College London, London, UK; 2 Camden and Islington NHS Foundation Trust, London, UK; 3 University College London Medical School, London, UK; 4 Sleep and Circadian Neuroscience Institute, University of Oxford, Oxford, UK; 5 Department of Statistical Science, University College London, London, UK

**Keywords:** dementia, actigraphy, aging; insomnia

## Abstract

**Study Objectives:**

Sleep disturbances are a feature in people living with dementia, including getting up during the night, difficulty falling asleep, and excessive daytime sleepiness and may precipitate a person with dementia moving into residential care. There are varying estimates of the frequency of sleep disturbances, and it is unknown whether they are a problem for the individual. We conducted the first systematic review and meta-analysis on the prevalence and associated factors of sleep disturbances in the care home population with dementia.

**Methods:**

We searched Embase, MEDLINE, and PsycINFO (29/04/2019) for studies of the prevalence or associated factors of sleep disturbances in people with dementia living in care homes. We computed meta-analytical estimates of the prevalence of sleep disturbances and used meta-regression to investigate the effects of measurement methods, demographics, and study characteristics.

**Results:**

We included 55 studies of 22,780 participants. The pooled prevalence on validated questionnaires of clinically significant sleep disturbances was 20% (95% confidence interval, CI 16% to 24%) and of any symptom of sleep disturbance was 38% (95% CI 33% to 44%). On actigraphy using a cutoff sleep efficiency of <85% prevalence was 70% (95% CI 55% to 85%). Staff distress, resident agitation, and prescription of psychotropic medications were associated with sleep disturbances. Studies with a higher percentage of males had a higher prevalence of sleep disturbance.

**Conclusions:**

Clinically significant sleep disturbances are less common than those measured on actigraphy and are associated with residents and staff distress and the increased prescription of psychotropics. Actigraphy appears to offer no benefit over proxy reports in this population.

**Statement of Significance**

Our findings show that 20% of care home residents with dementia are having clinically significant sleep problems when measured on validated informant questionnaires, but that this goes up to 70% when sleep disturbances are measured using actigraphy. This highlights that the prevalence of sleep disturbances varies greatly depending on how they are measured, highlighting the need for improvement of measurement in this population. In addition, sleep disturbances seem to be more common in men with dementia. These disturbances do seem to affect the individuals themselves with dementia in terms of being related to increased prescriptions of psychotropic medications and agitation, and they distress the staff who care for them, and therefore need evidence-based recommendations on how they should be managed.

## Introduction

There are currently 50 million people worldwide living with dementia, and this is projected to increase to 152 million by 2050 [1]. People living with dementia often have sleep disturbances including difficulty falling asleep, nighttime awakening and wandering, and excessive daytime sleepiness [2]. Sleep disturbances impact family carers, who report that being woken during the night is the most distressing sleep disturbance [3]. Families may be unable to continue caring at home, and thus people with dementia who have disturbed sleep are more likely to move into a care home [4, 5], which in turn increases the cost of care [6].

Sleep disturbances, therefore, may be highly prevalent in people with dementia who live in care homes, although individual studies vary markedly in their findings. A previous systematic review on the prevalence of neuropsychiatric symptoms in dementia [7] included three studies of sleep disturbance prevalence in nursing homes [8–10], but no meta-analysis has been conducted. A second systematic review and meta-analysis reported the prevalence of sleep disturbances in people with Alzheimer’s disease as 39% when measured via validated questionnaires [11]; however, most participants in the included studies lived in the community.

Sleep disturbances in people with dementia are often measured by validated proxy questionnaires as dementia can impact an individual’s ability to accurately recall their sleep, particularly in the care home population where dementia is often more severe than in the community [12]. More recently, actigraphy has been used where an accelerometer, typically worn on the wrist, measures the intensity of body movement to infer sleep and wake states [13]. There is no consensus on the best way to measure sleep disturbances in people with dementia, with previous studies comparing both methods in community-dwelling people with dementia and finding differing results [14, 15]. Comparing them may help to illuminate their meaning when measuring sleep disturbances in the care home population with dementia.

There is, to our knowledge, no previous systematic review focusing on sleep disturbances in the care home population with dementia. Thus, we aimed to produce the first systematic review and meta-analysis of the prevalence of sleep disturbances in people with dementia living in care homes and to explore what factors are associated with these sleep disturbances.

## Method

### Search strategy and selection criteria

We followed the PRISMA guidelines [16] and registered the protocol on PROSPERO (CRD42017080312). We searched Embase, MEDLINE, and PsycINFO from database inception to November 2, 2017, and updated the search until April 29, 2019. We used the search terms (“Dementia” OR “Alzheimer*”) AND (“sleep*” OR “insomnia” OR “circadian” OR “night*” OR “neuropsychiatric”) AND (“care home*” OR “residential” “home*” OR “nursing home*” OR “residential care” OR “long-term care” OR “long term care” OR “institution”), with no restriction on language. We searched reference lists of included papers and relevant systematic reviews and emailed authors of included papers for further relevant papers.

We included quantitative studies that reported:

□ the prevalence of, or factors associated with, sleep disturbances in people with dementia living in care homes;
□ results reported separately if they included community-dwelling people with dementia or people without dementia;
□ sleep disturbances by validated questionnaires or actigraphy measures (e.g. nighttime sleep efficiency);
□ cross-sectional data (in longitudinal studies we used baseline data only).

We excluded studies if:

□ sleep disturbances were an inclusion or exclusion criteria;
□ study participants were reported as having sleep-related breathing or movement disorders rather than sleep disturbances;
□ the study reported only rest activity or circadian rhythm variables rather than sleep disturbances;
□ data were collected during admission to a care home.

We contacted authors if we needed additional data or information to include a study. We defined a care home as a long-term residential or nursing care facility in the community, which provides 24-hour personal or nursing care for people with illness, disability, or dependence [17]. We characterized sleep disturbances as any well-defined disturbance in the sleep process, including difficulty falling asleep, reduced duration of sleep, waking or getting up during the nighttime, and excessive daytime sleepiness [2].

One researcher (LW) screened all titles and abstracts, and two researchers (LW and AL or AS) independently screened full texts and reached a consensus on included papers. We extracted data including country, setting, study design, sample size, dementia type, dementia severity, how dementia was defined, mean age, percentage of females, measure of sleep disturbances, measures of potential associated factors, and reported statistical results of prevalence and/or associated factors, and if any analyses were adjusted.

Two researchers (LW and JBu) independently assessed the quality of studies using the Mixed Methods Appraisal Tool (MMAT)—Version 2011 [18] criteria for quantitative descriptive studies. This assesses studies on four elements with scores ranging from 0 to 4 and higher scores indicating higher quality studies:

Is the sampling strategy relevant to address the quantitative research question (standardized method of sampling)?
Is the sample representative of the population under study (e.g. whole care home population with dementia)?
Are measurements appropriate (clear origin, or validity known, or standard instrument)?
Is there an acceptable response rate (≥60%)?

### Data analysis

We separated prevalence data into three methods of measurement: (1) informant rated validated questionnaires for clinically significant cases of sleep disturbances, (2) informant rated validated questionnaire for any symptoms of sleep disturbances, and (3) actigraphy measured sleep disturbances. We used STATA version 14 and the Metaprop command [19] that uses inverse-variance weights to conduct random effect meta-analyses of pooled prevalence. We conducted meta-analyses separately for each category of measurement, calculated confidence intervals (CI) using the exact method [20], and used the *I*^2^ statistic to assess heterogeneity (≥75% indicating high heterogeneity).

We conducted post hoc random effects meta-regressions using the Metareg command to investigate if study characteristics and participant demographics could explain the high heterogeneity in prevalence estimates. We combined data from all three meta-analyses and explored the category of prevalence measurement as a single covariate, and then as the first covariate in a further five meta-regressions, which included looking at the study covariates of age, percentage of females, dementia type (Alzheimer’s disease vs mixed/not specified), publication year, and study quality.

We assessed publication bias in the studies where data were meta-analyzed using funnel plots. We deemed studies that did not have an acceptable response rate (of 60% and above as defined by the MMAT), or who did not report the response rate, as lower quality studies and used this as criteria for sensitivity analyses. We provide a narrative synthesis for factors associated with sleep disturbances reported in individual studies.

## Results

**Figure 1.**
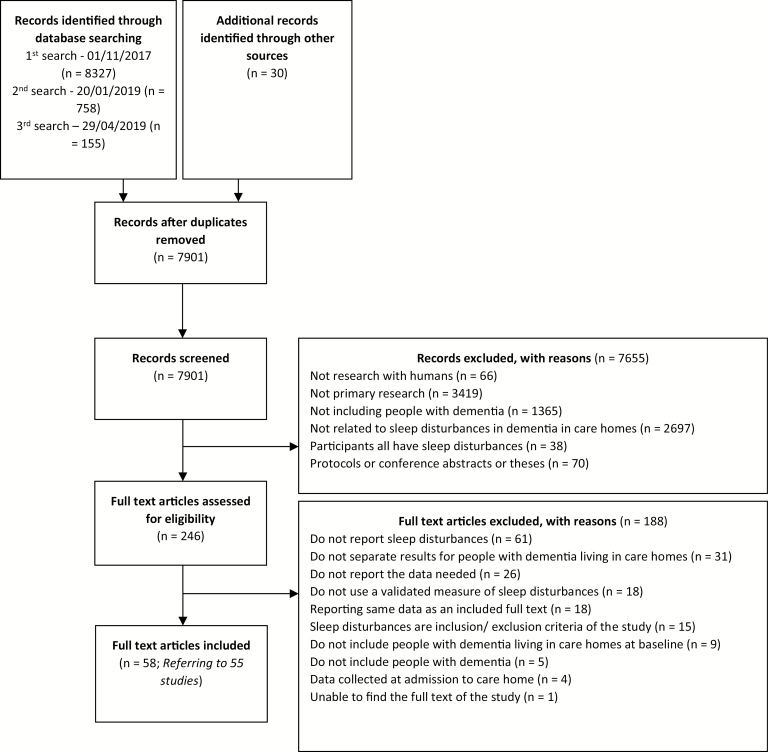
PRISMA flow diagram.

We screened 7901 references (Figure 1, PRISMA diagram), of which 58 papers comprising 55 studies met the inclusion criteria (Table 1). Fifty-one studies reported the prevalence of sleep disturbances [9, 10, 21–69], and 20 studies reported factors associated with sleep disturbances [24, 27, 30, 32, 35, 37, 38, 40, 41, 45, 49, 54, 61, 62, 64, 69–73]. Sixteen studies provided extra data when contacted [21, 28, 34–36, 39, 41, 47, 48, 50, 57–60, 62, 63]. Forty of the studies took place in Europe including studies in Denmark [60], France [26–28, 39], Germany [36, 47, 48, 64, 69], Italy [33, 43, 67], Netherlands [10, 22, 23, 30, 31, 45, 68], Norway [9, 21, 24, 25, 29, 34, 35, 44, 61], Poland [41, 71, 72], Portugal [50], Spain [32, 52], and Sweden [56, 57, 63, 66]. Others took place in Australia [42, 53, 54, 62], China [65, 73], Japan [40, 59, 70], South Korea [38, 49], Taiwan [37, 51], the United States [46], and Brazil [55].

**Table 1.** Study characteristics and quality ratings

Study reference	N (N sleep disturbances measured in)	Number of care homes	Dementia type	Dementia severity	Females (%)	Mean age	Measure of sleep disturbances	Study quality				
								1	2	3	4	Total
Aasmul et al. 2014 [35, 96]	352 (341)	18	Not specified	Advanced	74.4	86.0	NPI sleep item	✓	✘	✓	✓	3
Appelhof et al. 2019 [68]	274 (227)	13	All young onset; AD 43.8%; VaD 10.6%; FTD 29.2%; mixed AD/VaD 5.1%; LBD/PPD 1.8%; alcohol-related dementia 2.2%; other 7.3%	GDS mild 15.7%; moderate 20.8%; severe 62.8%	49.6	63.8	NPI sleep item	✓	✘	✓	?	2
Aupperle et al. 2004 [46]	173 (134)	29	All AD	Moderate to severe	81.5	82.6	NPI sleep item	✓	✘	✓	?	2
Balzotti et al. 2018 [67]	30 (30)	1	57% AD, 43% VaD	Mean MMSE score 7.6	83.3	85.7	NPI sleep item	✓	✘	✓	?	3
Bergh et al. 2011 [9]	169 (169)	7	Not specified	CDR mild 20.71%; moderate 37.27%; severe 42.01%	69.2	84.9	NPI sleep item	✓	✓	✓	✓	4
Bergh et al. 2012 [21]	620 (619)	32	Not specified	CDR mild 22%; moderate 29%; severe 50%	71.0	84.7	NPI sleep item	✓	✓	✓	✓	4
Bidzan et al. 2006 [71]	31 (31)	2	All AD	Mean MMSE score 14.8	Not specified	79.2	NPI sleep item	✓	✘	✓	✓	3
Bidzan et al. 2008 [72]	58 (58)	3	All AD	MMSE score between 11 and 23	Not specified	77.0	NPI sleep item	✓	✘	✓	✓	3
Bidzan et al. 2014 [41]	48 (48)	1	All AD	Mean MMSE score 15.96	Not specified	70.0	NPI sleep item	✓	✘	✓	?	2
Bjork et al. 2018 [63]	2827 (2827)	not specified	Not specified	Mild 37.7%; moderate 38.6%; severe 23.6%	69.9	85.6	NPI sleep item	✓	✓	✓	✓	4
Blytt et al. 2017 [24]	1535 (1535)	64	Not specified	Mild 35%; moderate 29%; severe 36%	75.7	85.3	NPI sleep item	✓	✓	✓	✓	4
Blytt et al. 2018 [25]	106 (106)	47	Not specified	Mean MMSE score 7.6	76.0	85.5	Actigraphy	✓	✘	✓	✓	3
Boada et al. 2006 [52]	79 (79)	2	All AD	Mild 26.6%; moderate 35.4%; moderately severe 19%; severe 19%	73.4	83.7	BEHAVE-AD diurnal rhythm disturbance item	✓	✓	✓	?	3
Brodaty et al. 2001 [53]	505 (505)	11	All AD	Not specified	74.1	83.4	BEHAVE-AD diurnal rhythm disturbance item	✓	✓	✓	✓	4
Brown et al. 2015 [62]	22 (22)	4	Not specified	Not specified	73.0	85.6	Actigraphy	✓	✘	✓	✘	2
Castineiras et al. 2012 [32]	212 (212)	6	AD 26.9%; VaD 18.9%; mixed 7.1%; DLB 0.9%; FTD 0.5%; unknown 45.8%	Mild 14.6%; moderate 16.5%; moderately severe 35.4%; severe 33.5%	73.1	85.7	NPI sleep item	✓	✓	✓	?	3
Chen et al 2018 [65]	112 (112)	1	Not specified	CDR mild 10.7%; moderate 39.3%; severe 50.0%	63.4	81.2	NPI sleep item	✓	✘	✓	✘	2
Cheng et al. 2009 [51]	63 (63)	not specified	All AD	Mean MMSE 10.3	60.7	81.9	BEHAVE-AD diurnal rhythm disturbance	✓	✘	✓	?	2
Cunha et al. 1985 [55]	227 (227)	10	Not specified	Severe 77.5%; mild 22.5%	84.7	75.6	CGBRS sleep problem item	✓	✘	✓	✓	3
Dechamps et al. 2008 [26]	109 (109)	4	Not specified	MMSE ≥24 9%, MMSE between 10 and 23 61.5% MMSE<10 29.5%	76.1	83.0	NPI sleep item	✓	✘	✓	✓	3
Dichter et al. 2015 [48]	154 (154)	9	Not specified	FAST stages 2-6 63.6%; stage 7 36.4%	83.1	83.1	NPI sleep item	✓	✓	✓	✓	4
Ferreira et al. 2016 [50]	97 (97)	3	Not specified	Mean MMSE score 22	90.0	81.0	NPI sleep item	✓	✘	✓	✓	3
Fetveit et al. 2002 [61, 97]	29 (25)	1	Majority AD, number not specified	Mean MMSE score 13.4	not specified	85.4	Actigraphy	✓	✓	✓	✓	4
Gustafsson et al. 2016 [56]	3482 (3482)	not specified	Not specified	Mean GCS score 11.8 in 2007; 12.4 in 2013	69.4	84.8	MDDAS interrupted night sleep item	✓	✘	✓	✓	3
Hsieh et al. 2009 [37]	103 (103)	10	AD 50.5%; VaD 49.5%	Mean CDR score 1.38 AD; 1.33 VD	47.6	72.2	NPI sleep item	✓	✘	✓	?	2
Koopmans et al. 2009 [23]	39 (39)	2	70% AD; 10% VaD; 20% not specified	Advanced	90.0	83.0	NPI sleep item	✓	✘	✓	✓	3
Krolak‐Salmon et al. 2016 [28]	211 (211)	not specified	All AD	Not specified	61.1	84.0	NPI sleep item	✓	✘	✓	✓	3
Kume et al. 2016 [59]	17 (17)	4	AD 58.8%; VaD 41.2%	Mean CDR score 1.4	58.8	82.2	Actigraphy	✓	✘	✓	?	2
Lam et al. 2006 [73]	125 (125)	3	AD 43.2%; VaD 24.8%; not specified 32.0%	Not specified	58.4	82.0	NPI sleep item	✓	✘	✓	?	2
Lee et al. 2015 [38]	529 (529)	20	Not specified	Not specified	77.5	81.2	NPI sleep item	✓	?	✓	?	2
Lichtwarck et al. 2018 [34]	229 (229)	33	Not specified	Mild 4.4%; moderate 25.8; severe 69.5%	60.3	83.2	NPI sleep item	✓	✘	✓	✓	3
Lövheim et al. 2009 [57]	1826 (1826)	not specified	Not specified	Mean GCS score 11.5	68.9	82.8	MDDAS interrupted night sleep item	✓	✓	✓	✓	4
Malara et al. 2016 [43]	201 (201)	10	VaD 61.9%; AD 29.3%	Mild 11.1%; moderate 27.1%; severe 61.9%	66.3	83.9	NPI sleep item	✓	✓	✓	✓	4
Melander et al. 2018 [66]	14 (14)	5	VaD 50%, AD 14.3%, FTD 14.3%, mixed 14.3%, LBD 7.1%	All GDS score 6	78.6	81.5	NPI sleep item	✓	✘	✓	✓	3
Mulders et al. 2016 [22]	230 (225)	8	All young onset; AD 32.0%; VaD 12.9%; FTD 16.0%; AlcD 17.8%; Other 21.3%	GDS score 2-4 17.3%; score 5 24.4%; score 6 30.2%; score 7 28.0%	46.7	60.1	NPI sleep item	✓	✘	✓	✓	3
Ozaki et al. 2017 [40]	312 (200)	10	AD 35.9%; VaD 19.6%; other 9.9%; not specified 34.6%	Mild 28.8%; moderate 54.8%; severe 16.3%	82.4	87.6	NPI sleep item	✓	✓	✓	✘	3
Palm et al. 2018 [64]	1132 (1132)	140	Not specified	DSS mean score 9.5	79.2	83.4	NPI sleep item	✓	✓	✓	✘	3
Prado-Jean et al. 2010 [27]	319 (319)	17	Not specified	Mild 24.4%; moderate 50.2%; severe 25.4%	76.5	85.6	NPI sleep item	✓	✘	✓	✓	3
Reuther et al. 2016 [47]	840 (840)	40	Not specified	FAST scale mild 3.8%; moderate 63.5%; severe 32.7%	76.0	85.0	NPI sleep item	✓	✘	✓	?	2
Ricci et al. 2009 [33]	173 (157)	1	AD 44.5%; VaD 30.6%; mixed 17.3%; ns 10.8%; DLB 1.7%; PDD 1.2%; PPA 1.2%	Not specified	74.9	79.9	NPI sleep item	✓	?	✓	?	2
Ruths et al. 2008 [44]	55 (55)	13	Not specified	Not specified	78.2	84.1	NPI sleep item	✓	✘	✓	?	2
Schüssler et al. 2015 [58]	178 (178)	175	AD 52%; VaD 15.8%; other 19.2%; ns 13%	Mean MMSE 16.5	83.1	83.5	CDS Day-/night pattern item	✓	✘	✓	✓	3
Seidl et al. 2007 [36]	128 (128)	not specified	AD 77.3%; VaD or mixed 17.2%; Other 5.5%	GDS score ≤3 26%; score 4 14%; score 5 19%; score 6 30%; score 7 11%	81.4	84.8	NPI sleep item	✓	?	✓	?	2
Selbaek et al. 2014 [29]	931 (931)	26	Not specified	CDR 1 25%, 2 33%, 3 42%	74.0	84.5	NPI sleep item	✓	✓	✓	✓	4
Song et al. 2015 [49]	423 (423)	6	Not specified	Mild 9.1%; moderate 21.7%; severe 69.2%	82.0	83.3	NPI sleep item	✓	✘	✓	?	2
Suzuki et al. 2017 [70]	226 (226)	not specified	AD 47.0%; VaD 14.0%; LBD 1.0%; FTD 1.5%; mixed 15.5%; other 7.5%; not specified 13.5%	Mean MMSE score 9.53	76.6	85.1	NPI sleep item	?	✓	✓	✘	2
Tan et al. 2015 [54]	169 (169)	6	Not specified	Not specified	77.5	87.5	ESS	✓	✘	✓	✓	3
Thodberg et al. 2016 [60]	100 (70)	4	Not specified	Not specified	69.0	85.5	Actigraphy	✓	?	✓	?	2
Tournier et al. 2017 [39]	13 (11)	1	Not specified	36% moderate; 64% severe	90.9	82.9	NPI sleep item	✓	✓	✓	✓	4
Wetzels et al. 2010 [10]	290 (117)	9	AD 35.0%; VaD 11.1%; mixed AD/VaD 1.7%; other 52.1	GDS score 4 11.1; score 5 26.5; score 6 33.3; score 7 29.1%	71.7	81.7	NPI sleep item	✓	✘	✓	?	2
Wilfling et al. 2019 [69]	1187 (1187)	38	Not specified	Not specified	74.0	83.0	SDI	✓	✓	✓	✓	4
Wu et al. 2009 [42]	93 (93)	7	Not specified	GDS score 4 2.2%; Score 5 12.9%; score 6 55.9%; score 7 29.0%	76.3	88.6	NPI sleep item	✓	✘	✓	✘	2
Zuidema et al. 2006 [31]	59 (59)	2	Not specified	Not specified	83.0	82.0	NPI sleep item	✓	✘	✓	✓	3
Zuidema et al. 2007 [30, 98]	1437 (1437)	27	Not specified	Mild 4%; moderate 20%; moderately severe 51%; severe 26%	81.0	83.0	NPI sleep item	✓	✓	✓	✓	4
Zwijsen et al. 2014 [45]	432 (432)	17	AD 47.7%; VaD 19.0%; mixed AD/VaD 15.5%; DLB 3.7%; FTD 2.5%; other 8.6%	GDS score ≤3 1%; score 4 4%; score 5 21%; score 6 62%; score 7 12%	69.9	83.3	NPI sleep item	✓	✓	✓	✓	4

AD, Alzheimer’s Disease; CGBRS, Crichton Geriatric Behavioural Rating Scale; DSS, Dementia Screening Scale; ESS, Epworth Sleepiness Scale; FAST, Functional Assessment Staging Test; FTD, Frontotemporal dementia; GCS, Gottfries Cognitive Scale; GDS, Global Deterioration Scale; LBD, Lewy Body dementia; MMSE, Mini Mental State Examination; NPI, Neuropsychiatric Inventory; PDD, Parkinson’s disease dementia; PPA, Primary progressive aphasia; SDI, Sleep Disorder Inventory; VaD, Vascular dementia.

The majority of studies (*n* = 45) used the Neuropsychiatric Inventory (NPI) [74] sleep item, which measures sleep disturbances during the nighttime and excessive daytime sleepiness. Clinically significant cases are those who score ≥4 for the frequency times severity of the item [75]. Five studies [21, 34–36, 65] reported estimates of prevalence of both clinically significant cases and symptoms on the NPI and were included in two meta-analyses. One study used the Sleep Disorders Inventory [76], which is based on the NPI sleep item and the item subquestions.

Other measures used include the Epworth Sleepiness Scale (ESS) [77], a scale used in many different populations that reports daytime sleepiness and defines a clinically significant case by a score of ≥10 [78]. There were three measures of nighttime sleep disturbances (Behavioural Pathology in Alzheimer’s Disease [79] diurnal rhythm disturbance item, Multi-Dimensional Dementia Assessment Scale [80] interrupted night sleep item, and Crichton Geriatric Behavioural Rating Scale [81] sleep item) and one measure of sleep disturbances during the day and at night (Care Dependency Scale [82] Day/night pattern item).

Five studies measured sleep disturbances via wrist worn actigraphy; measures recorded included time spent asleep and awake at night, and sleep efficiency, which is the percentage of time spent asleep of the total time spent in bed. Sleep disturbance is often defined by a sleep efficiency of <85% [83–86]. In the five included studies, sleep efficiency was averaged over the nights the acti-watch was worn for, which varied from 1 night, 3 nights, 7 nights (in 2 studies), and 14 nights.

### Study quality

Quality scores across studies on the MMAT ranged from 2 to 4, out of a possible 4 (see Table 1). Thirteen studies were of higher quality scoring 4, 23 studies scored 3, and 19 studies scored 2. Nineteen studies did not report the proportion of responders, and for five studies, <60% of potential participants participated.

### Prevalence of sleep disturbances

**Figure 2.**
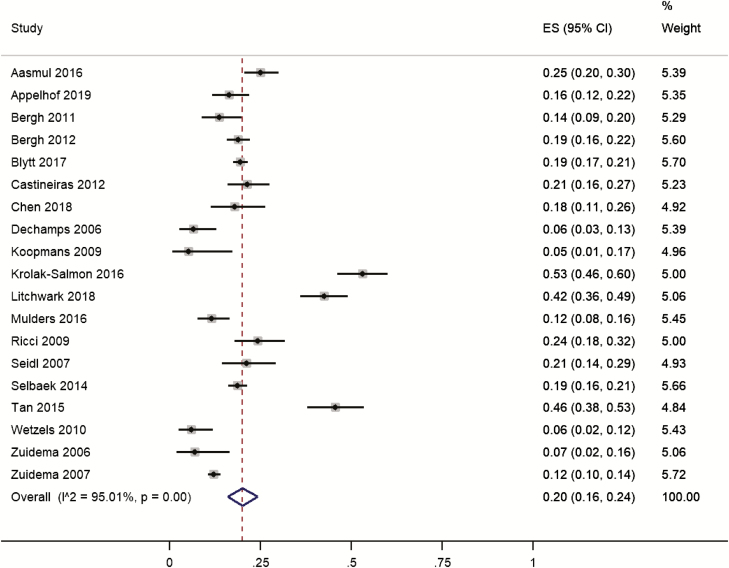
Forest plot of the prevalence of clinically significant sleep disturbances in people with dementia living in care homes measured by validated questionnaires. ES, effect size.

Nineteen studies on 7,026 participants reported the prevalence of clinically significant cases from validated questionnaires. Individual study prevalence ranged from 5% to 53%. Pooled prevalence was 20% (95% CI 16% to 24%; Figure 2). Heterogeneity was high (*I*^2^ = 95%). One of the studies reported only daytime sleepiness [54], whereas the others reported both excessive daytime sleepiness and nighttime sleep disturbances. Removing this study did not markedly alter the pooled estimate (19%; 95% CI 15% to 23%). We conducted a sensitivity analysis by removing six lower quality studies [10, 32, 33, 36, 65, 68], as assessed by MMATS, but the pooled estimate prevalence remained essentially unchanged (21%; 95% CI 16% and 26%).

**Figure 3.**
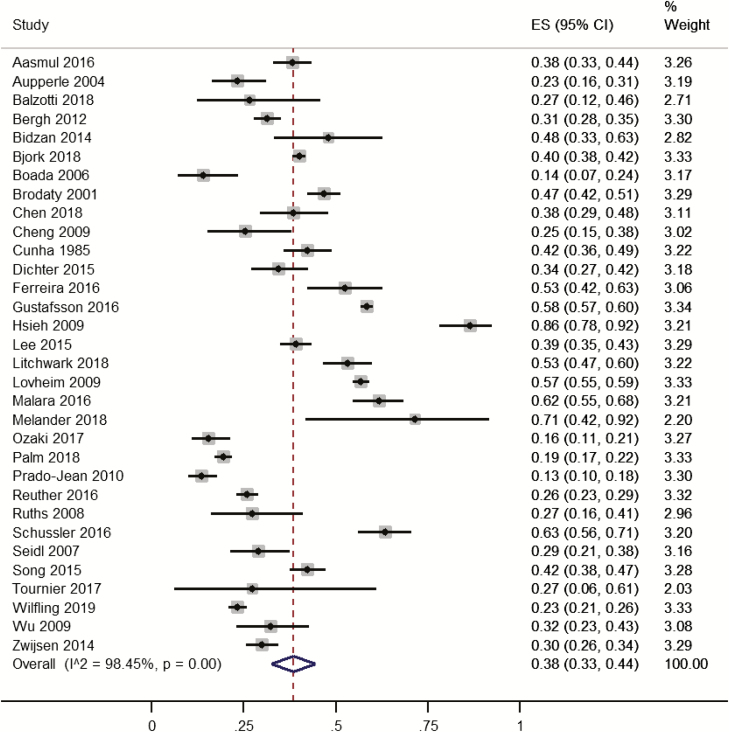
Forest plot of the prevalence of symptoms of sleep disturbances in people with dementia living in care homes measured by validated questionnaires. ES, effect size.

Thirty-two studies on 16,503 participants reported the presence of any sleep symptoms on validated questionnaires. Individual study prevalence ranged from 13% to 86%. Pooled prevalence was 38% (95% CI 33% to 44%; Figure 3). Heterogeneity was high (*I*^2^ = 98%). In sensitivity analysis, 15 lower quality studies [36–38, 40–42, 44, 46, 47, 49, 51, 52, 64, 65, 67] were removed and pooled prevalence increased slightly from 38% to 43% (95% CI 36% to 51%).

**Figure 4.**
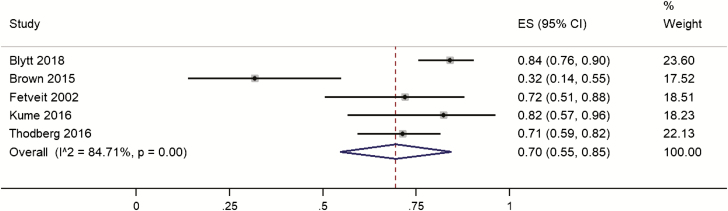
Forest plot of the prevalence of sleep disturbances on actigraphy in people with dementia living in care homes measure. ES, effect size.

Five studies on 240 participants reported sleep disturbances as measured by a sleep efficiency of <85% on actigraphy. Across the individual studies prevalence ranged from 32% to 84%. Pooled prevalence was 70% (95% CI 55% to 85%; Figure 4). Heterogeneity was high (*I*^*2*^ = 84%). Three studies [59, 60, 62] of lower quality were removed in sensitivity analysis and the pooled prevalence increased to 82% (95% CI 76% to 89%).

Meta-regressions showed that the method of measurement employed was a statistically significant moderator of prevalence (*F*_2,48_ = 16.00, *p* < 0.0001), with estimates of prevalence markedly increasing from clinically significant cases, symptoms, and then on actigraphy. After taking the method of measurement into account pooled meta-regressions also revealed that a higher percentage of males was associated with higher estimates of prevalence of sleep disturbances (*t*_48_ = −2.42, *p* = 0.020), though neither year of publication, study quality, average age of participants, or dementia type moderated the estimates of prevalence (all *p* > 0.10). We investigated publication bias by funnel plots for each meta-analysis, all of which appeared asymmetrical (Supplementary material Figures S1–S3), which could indicate publication bias.

### Associated factors

Overall, six factors were tested for association in more than one of the included studies (Table 2). Increased staff distress was overall consistently associated with sleep disturbances as measured on questionnaires, with three studies finding evidence for an association between sleep disturbances and staff distress in both nurses and care workers [35, 45, 49]. For residents, being agitated, including subtypes of verbal and physical agitations, was also consistently associated with having sleep disturbances reported on questionnaires [64, 71, 73]. When measured on actigraphy, having sleep disturbances was associated with physical agitation, but there was no evidence of an association with verbal agitation [62].

**Table 2.** Associates of sleep disturbance (considered in >1 study)

Factor	Measure of sleep disturbances	Study	Factors investigated for association with sleep disturbances	Number of significant relationships/times measured (%)
		[35]	Nurses and care workers distress (sleep disturbance mean score**)	
Staff distress about sleep disturbance	NPI	[49]	Nurses distress (sleep disturbance severity**) Nurse distress (sleep disturbance symptoms) Care worker distress (sleep disturbance symptoms** and severity**)	6/8 (75%)
		[45]	Staff distress in nurses (sleep disturbance severity,* mean score,* and frequency)	
Resident agitation	NPI	[71]	Agitation (sleep disturbance frequency* and severity*) Agitation—physical nonaggressive (sleep disturbance frequency* or severity*) Agitation—verbal aggressive (sleep disturbance frequency* or severity*) Agitation—physical aggressive (sleep disturbance frequency* or severity*) Agitation—verbal nonaggressive (sleep disturbance frequency or severity)	13/15 (87%)
		[73]	Incidence of challenging behaviors (Sleep mean score*) Frequency of challenging behaviors (Sleep mean score*) Difficulty of challenging behaviors (Sleep mean score*) Total challenging behaviors (Sleep mean score*)	
		[64]	Agitation (Sleep symptoms**)	
	Actigraphy	62	Agitation—physical nonaggressive (amount of nighttime sleep*) Agitation—verbal aggressive or nonaggressive (amount of nighttime sleep)	1/2 (50%)
Psychotropics	NPI	[35]	Antipsychotics (clinically significant sleep disturbance**) Antidepressants (clinically significant sleep disturbance*)	7/10 (70%)
		[32]	Antipsychotics (clinically significant sleep disturbance)	
		[38]	Antipsychotics (sleep disturbance symptoms or severity)	
		[30]	Any psychotropic (clinically significant sleep disturbance*) Hypnotics/sedatives (clinically significant sleep disturbance*) Antipsychotics (clinically significant sleep disturbance*) Anxiolytics (clinically significant sleep disturbance*) Antidepressants (clinically significant sleep disturbance)	
	SDI	[69]	Any psychotropic (sleep disturbance symptoms)	
Resident age	NPI	[32]	Age (clinically significant sleep disturbance)	0/3 (0%)
		[70]	Age (sleep disturbance mean score)	
	SDI	[69]	Age (sleep disturbance symptoms)	
Resident sex	NPI	[32]	Sex (clinically significant sleep disturbance)	1/2 (50%)
	SDI	[69]	Male sex (sleep disturbance symptoms*)	
Dementia severity	NPI	[72]	More severe dementia (sleep disturbance frequency* or severity*)	2/4 (50%)
		[32]	Dementia severity (clinically significant sleep disturbance)	
		[70]	Dementia severity (sleep disturbance mean score)	
	Actigraphy	[61]	Less severe dementia (duration of nighttime awakenings*) Dementia severity (% of sleep efficiency) Dementia severity (Amount of nighttime sleep)	1/3 (33%)

NPI, Neuropsychiatric Inventory; SD, sleep disturbance; SDI, Sleep Disorders Inventory.

* *p* < 0.05; **p < 0.001.

For psychotropic medications, overall, there was consistent evidence for an association with sleep disturbances reported on validated questionnaires; however, the evidence for individual psychotropics was mixed. In two studies, the prescription of antipsychotics was associated with having sleep disturbances [30, 35]; however, in two other studies, there was no evidence of an association [32, 38]. Similarly, antidepressants were associated with sleep disturbances in one out of two studies [30, 35]. Taking any psychotropic medication was associated with increased sleep disturbances in two studies [30, 69], as were hypnotics/sedatives, or anxiolytics in one study [30]. Resident sex had mixed results for an association with sleep disturbances, with an association with more males and increased prevalence of sleep disturbances [69], and no evidence for an association in one study [32].

The evidence for an association between dementia severity and sleep disturbances was mixed, both when measured by questionnaires and on actigraphy. On questionnaires more severe dementia was associated with more severe and frequent sleep disturbances in one study [72], but there is no evidence of an association in two other studies [32, 70]. On actigraphy, less severe dementia was associated with an increased duration of nighttime awakenings, but dementia severity was not associated with percentage of sleep efficiency or amount of nighttime sleep [61]. In three studies, age was not associated with sleep disturbances [32, 69, 70]. No other associated factors were reported across more than one study (all associates reported in Supplementary Table S1).

## Discussion

This is the first systematic review and meta-analysis investigating the measurement and prevalence of sleep disturbances in people with dementia living in care homes. We found that the pooled prevalence of clinically significant sleep disturbance was 20%; this was less common than having any symptom of sleep disturbance, which occurred in 38%. Actigraphy-determined sleep disturbance was much higher (70%). In meta-regressions, the method of sleep disturbance measurement was a highly statistically significant moderator of outcome, and the confidence intervals for the different methods did not overlap. It seems that these different methods are measuring different phenomena, or potentially different groups of people living in care homes.

In addition, the percentage of males within a study was important, as a higher percentage of males was associated with a higher prevalence of sleep disturbances, and this association was also found in one of the individual studies [69]. This finding was robust to adjustment by method of measurement. There were a variety of other demographic and illness related factors tested within individual studies for their association with sleep disturbances with overall consistent findings for staff distress, resident agitation and prescription of psychotropic medications.

A previous meta-analysis investigated questionnaire rated prevalence of sleep disturbances in people with Alzheimer’s disease, most of whom lived in the community [11]. Of the studies included in the previous meta-analysis, most (16/17) measured sleep as any symptoms of sleep disturbance, with one study measuring clinically significant sleep disturbances. They found a pooled estimate of 39%, similar to the figure found in our meta-analysis of symptoms of sleep disturbance in care homes.

We found that the prevalence of sleep disturbances varied greatly dependent on the measurement method, and disagreements between actigraphy and questionnaires has been found in previous studies of people with dementia living in the community [14, 15, 87–89]. A recent cross-sectional study compared reports of sleep disturbances on proxy questionnaires with actigraphy in care home residents with and without dementia [85]. Similar to our findings, they found that 20.5% of residents were classified as having clinically significant cases of sleep disturbance on the NPI sleep item, and that 89.2% of the same residents had a sleep efficiency of less than 85% on actigraphy. The authors of that direct comparison argue that the large discrepancies in rates of sleep disturbance between actigraphy and proxy questionnaires implies that care home staff are unaware of many residents being disturbed during the night, and people are not receiving treatment when they should be [85]. However, questionnaires report broader sleep disturbances than actigraphy, such as daytime sleepiness, and when answered by an informant they reflect the impact of sleep disturbance on both family and paid carers.

On the other hand, actigraphy may overestimate sleep disturbances. As people get older sleep efficiency significantly decreases, with a 3% decrease every decade of age [90]. Therefore, it is possible that a sleep efficiency threshold of 85% that was developed in healthy adults [83–86], may not be applicable to older adults who have dementia, though it is still used. Residents in care homes often spend a long time in bed over the nighttime [85, 91], which could also lead to lower sleep efficiency without sleep being disturbed as the sleep window, the period between when someone goes to bed and when they get up to start the day, is longer. One of the studies mentioned that residents could decide their bedtime, but rising time was influenced by the care homes routine [61], so someone going early to bed and then waking before the staff helped them get ready for the day could have been classed as sleep disordered. However, spending an extended time in bed itself often fragments and disturbs sleep [92]. Similarly, care home residents may spend some of the daytime napping, which could also fragment sleep as the nocturnal drive for sleep is reduced [25, 93]. Of the five actigraphy studies included in this review, three were of lower quality, which may also account for some of the differences in prevalence estimates between actigraphy and questionnaires.

Inclusion criteria for participating in an actigraphy study were generally more restrictive than for other measurement methods, which could have biased the discrepancies in prevalence between questionnaires and actigraphy. However, we do not think this potential bias is likely to account for the significant differences in prevalence of sleep disorders between actigraphy and clinical questionnaires. This is because the actigraphy studies excluded those with severe aggression or pain [25], immobile and bed-bound participants, as they could not define rising and bedtime for these people [25, 61], or those who had been recently hospitalized [59, 62], or used benzodiazepines within 1 month [59]. This more severely ill population would be likely to have had a higher level of sleep disturbances, so that its exclusion in the actigraphy studies would have potentially led to a lower, not higher, prevalence.

We found that a higher percentage of men living in a care home was associated with a higher prevalence of sleep disturbances in this population. It is unknown whether this finding might be associated with concurrent additional neuropsychiatric symptoms that might differ between men and women. A previous meta-analysis that found no sex differences in the prevalence of sleep disturbances in people with dementia living the community [11] also found no sex differences in the prevalence of other neuropsychiatric symptoms on the NPI. Additional studies are needed to examine this issue.

We also explored whether age, publication year, study quality, and dementia subtype influenced the prevalence of sleep disturbances, though we found these characteristics did not. With dementia subtypes, the accuracy of the diagnoses can be unreliable [94], and most of the included studies did not specify dementia type, hence why we compared studies with only Alzheimer’s disease compared to mixed or not specified.

Sleep disturbances were also associated with increased prescription of psychotropics across individual studies. Residents in the studies may be receiving psychotropic medication for reasons that could be contributing to the development of sleep disturbances, such as anxiety, depression, or psychosis. Similarly, as studies tended to record what medications were prescribed, and not what medications were taken, this may be unreliable information. Sleep disturbances were also associated with increased agitation, but it is unclear if agitation is a cause or consequence, or potentially both, of sleep disturbances.

There was high heterogeneity in the estimates of prevalence across individual studies, which may be explained because included studies were heterogeneous in several aspects: they had been published across many years, in various countries with varying admission criteria for care homes, and using different study designs. One factor that varied substantially was the sample size of included studies, and questionnaire studies often had larger samples. Those using questionnaires ranged from 11 to 3,482 participants, to those using actigraphy ranging from 17 to 106 participants. Studies also used different measures of sleep disturbances. In one study, a small minority (9.3%) of residents with dementia self-reported their daytime sleepiness via the ESS [54]; in all other studies, a care home staff member reported sleep disturbances. Some studies had stricter exclusion or inclusion criteria, e.g. excluding those with a life expectancy of less than 6 months, or only including those with clinically significant agitation or those referred for management of neuropsychiatric symptoms, which may further explain the heterogeneity within the estimates across individual studies.

### Strengths and weaknesses of the review

To our knowledge, this is the most comprehensive systematic review to date on the prevalence of sleep disturbances in dementia. We systematically searched three databases and contacted the authors of included studies for further papers and additional data. We were consequently able to add 16 studies providing unpublished reports of prevalence. However, we only included published studies and did not search the gray literature. While two reviewers screened all full texts for inclusion, and agreements were reached by consensus, only one reviewer screened all abstracts and titles. We had no restriction on language and included nine studies published in languages other than English, including studies taking place across five continents. A limitation of our review is that we only used cross-sectional baseline data from all studies; therefore, longitudinal changes in the prevalence of sleep disturbances and the causal mechanisms of any significantly associated factors are unclear. Many studies did not adjust for confounding variables in analyses of associated factors, and studies may have been less likely to report nonsignificant associations.

### Treatment implications of our findings

As our findings indicate a large discrepancy between prevalence by method of measurement, this could have implications for if sleep disturbances are treated as actigraphy may classify an individual as having a sleep problem when a questionnaire does not, or vice versa. For example, actigraphy may be overestimating sleep problems, and this could lead to care home residents with dementia being treated for disturbances that they do not have. This could have further implications as hypnotic medications prescribed for sleep disturbances can increase risk of falls and other undesirable outcomes in this population [95] and would have no benefit for those who are wrongly classified as sleep disturbed. However, on the other hand, questionnaires may be underestimating sleep problems in this population, possibly because care home staff may not always know someone is awake, and therefore residents may not be adequately treated for these disturbances that could be having a negative effect on them.

In conclusion, sleep disturbances are prevalent in care home residents with dementia, with large discrepancies between estimates of prevalence on validated questionnaires and on actigraphy. Those seem to be measuring different concepts of disturbed sleep. It is important that sleep disturbances are measured accurately as identification is necessary for treatment. Future research is needed to understand the precision of actigraphy and questionnaires in people with dementia. Questionnaires are currently advantageous as they are quicker, cheaper, and more feasible to measure sleep disturbances in all residents with dementia and are clinically important as they measure a phenomenon associated with resident’s agitation, being prescribed psychotropic drugs and staff distress. Further longitudinal research is needed to illustrate the direction of these associations.

## Supplementary Material

zsz251_suppl_Supplementary_Material
