# Supplementary material for: Measuring the prevalence of sleep disturbances in people with dementia living in care homes: a systematic review and meta-analysis
Source: Sleep. 2019 Oct 21;43(4):zsz251. doi: 10.1093/sleep/zsz251 (PMC7157185; doi:10.1093/sleep/zsz251)
Supplement: zsz251_suppl_Supplementary_Material [file zsz251_suppl_supplementary_material.docx]

***Supplementary data and analyses***


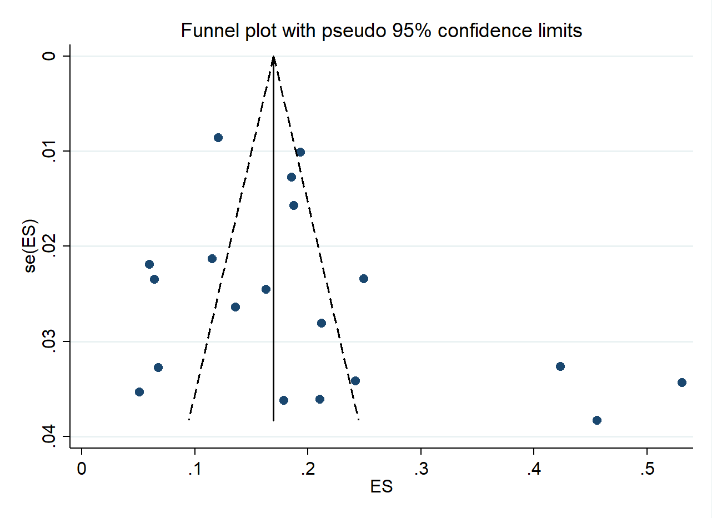


***Figure S1:* funnel plot for studies reporting the prevalence of clinically significant cases of sleep disturbances (ES = effect size, se(ES) = standard error of effect size)**


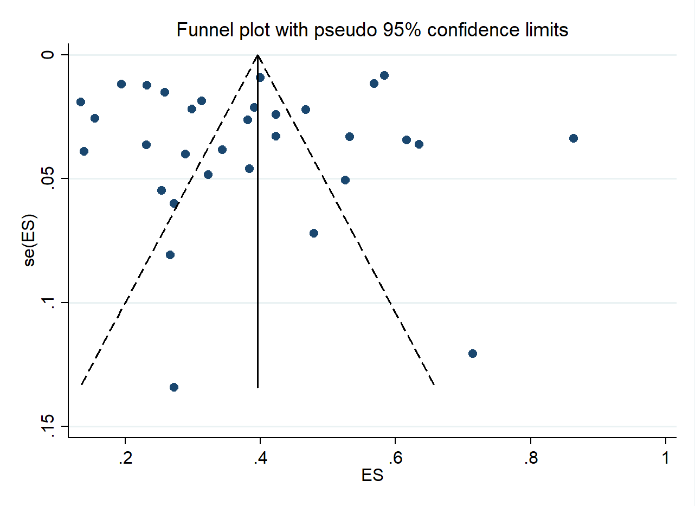


***Figure S2:* funnel plot for studies reporting the prevalence of symptoms of sleep disturbances (ES = effect size, se(ES) = standard error of effect size)**


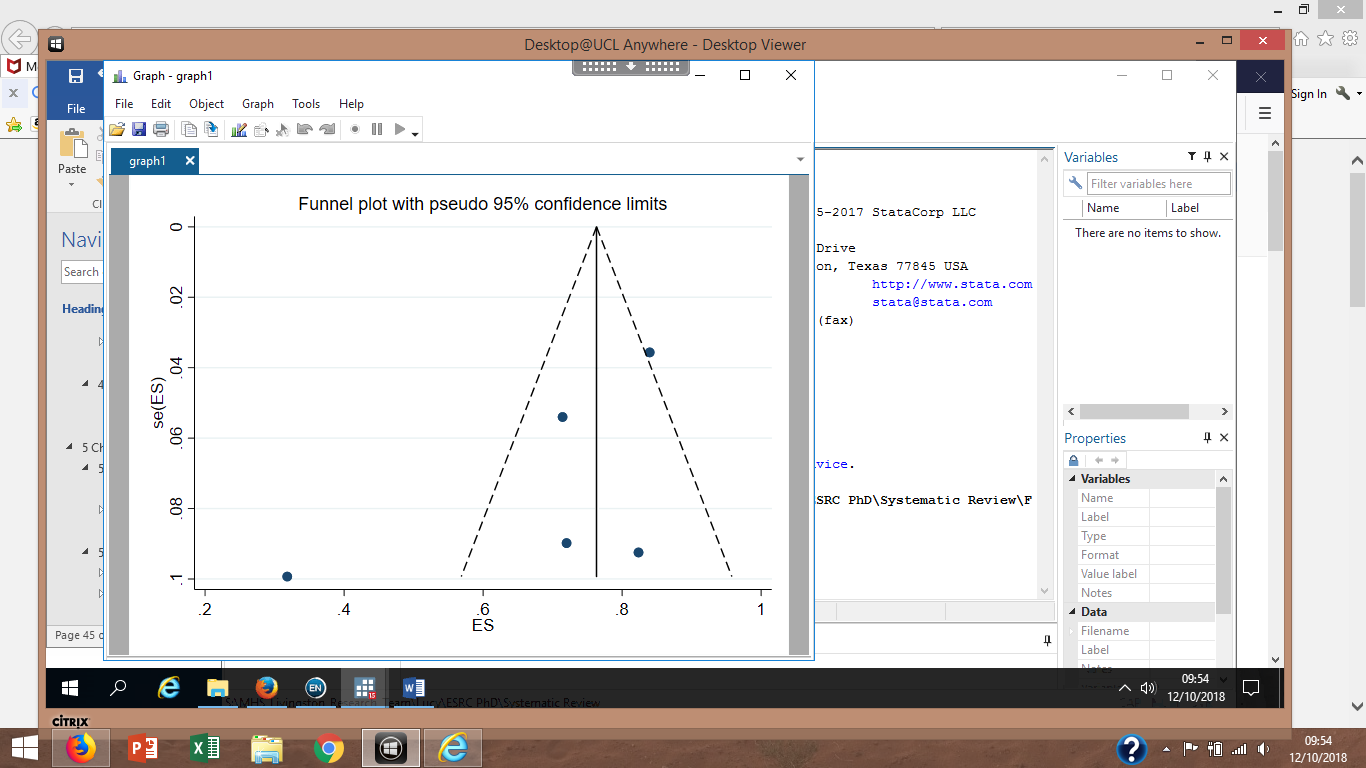


***Figure S3:* funnel plot for studies reporting the prevalence of sleep disturbances on actigraphy (ES = effect size, se(ES) = standard error of effect size)**

***Table S1: Factors that are and are not associated with sleep disturbances***

| Study | Type of measure of sleep disturbances | Factors significantly associated | Statistic | Factors not significantly associated | Statistic |
| --- | --- | --- | --- | --- | --- |
| Aasmul et al 2016^35, 87^ | Clinically significant cases (NPI sleep item; any clinically significant symptom) | + antipsychotics  + antidepressants  + anxiety  + pain | p<0.001  p=0.040  p<0.001  p=0.032 | - | - |
| Aasmul et al 2016^35, 87^ | Symptoms (NPI sleep item; mean score) | + staff distress | p<0.001 | - | - |
| Bidzan et al 2006^71^ | Symptoms (NPI sleep item; frequency, severity) | + agitation  + physical non-aggressive agitation  + verbal aggressive agitation  + physical aggressive agitation | p<0.05 (frequency); p<0.05 (severity)  p<0.05 (frequency); p<0.05 (severity)  p<0.05 (frequency); p<0.05 (severity)  p<0.05 (frequency); p<0.05 (severity) | + agitation verbal non-aggressive subscale | p>0.05 (frequency); p>0.05 (severity) |
| Bidzan et al 2008^72^ | Symptoms (NPI sleep item; frequency, severity) | + dementia severity | p<0.05 (frequency); p<0.05 (severity) | - | - |
| Bidzan et al 2014^41^ | Symptoms (NPI sleep item; frequency, severity, mean score) | - | - | + vascular component of AD | p>0.05 (frequency); p>0.05 (severity); p>0.05 (mean score) |
| Blytt et al 2017^24^ | Symptoms (NPI sleep item; any symptoms) | - | - | + cancer | p>0.05 |
| Brown et al 2015^62^ | Actigraphy (amount of night-time sleep) | + physical non-aggressive agitation | p=0.037 | + agitation – verbal | p=0.319 |
| Castineiras et al 2012^32^ | Clinically significant cases (NPI sleep item; any clinically significant symptom) | + cholinesterase inhibitors/memantine  + months living in a care home | p<0.05  p<0.05 | sex  + age  + dementia severity  + antipsychotics | p>0.05  p>0.05  p>0.05  p>0.05 |
| Fetveit et 61, 88 | Actigraphy (sleep efficiency, duration of night time awakenings, amount of night-time sleep) | - dementia severity | p<0.05 (duration of night-time awakenings) | + dementia severity | p=0.12 (% of sleep efficiency); p=0.25 (Amount of night-time sleep) |
| Hsieh et al 2009^37^ | Symptoms (NPI sleep item; any symptom, mean score) | AD (vs VaD) | p<0.05 (presence) | AD (vs VaD) | p>0.05 (mean score) |
| Lam et al 2006^73^ | Symptoms (NPI sleep item; mean score) | + incidence of challenging behaviours  + frequency of challenging behaviours  + difficulty of challenging behaviours  + total challenging behaviour score | p<0.01  p<0.01  p<0.01  p<0.01 | - | - |
| Lee et al 2015^38^ | Symptoms (NPI sleep item; any symptom, severity) | - | - | + Antipsychotics | p=0.508 (presence); p=0.406 (severity) |
| Palm et al 2018^64^ | Symptoms (NPI sleep item; any symptom) | + agitation | p<0.001 | - | - |
| Prado-Jean et al 2010^27^ | Symptoms (NPI sleep item; any symptom, mean score) | + depression  + depression (only those with moderate dementia)  + depression (only those with severe dementia) | p<0.0001 (presence); p<0.0001 (mean score)  p=0.002 (presence)  p=0.005 (presence) | + depression (only those with mild dementia) | p=0.114 (presence) |
| Song et al 2015^49^ | Symptoms (NPI sleep item; any symptom, severity) | + staff distress in nurses  + staff distress in care workers | p<0.001 (severity)  p<0.001 (presence); p<0.001 (severity) | + staff distress in nurses | p>0.05 (presence) |
| Suzuki et al 2017^70^ | Symptoms (NPI sleep item; mean score) | - quality of life (experiencing minimum negative behaviours subscale) | p=0.000 | + dementia severity  + age  + quality of life (interacting with surroundings subscale)  + quality of life (expressing self subscale)  + impairment in activities of daily living | p=0.754  p=0.074  p=0.682  p=0.180  p=0.631 |
| Tan et al 2015^54^ | Clinically significant cases (ESS; any clinically significant symptom) | - | - | + paracetamol use  + opioid use  + analgesic load >0-<4  + analgesic load ≥4 | p=0.145  p=0.691  p=0.553  p=0.336 |
| Wilfling et al 2019^69^ | Symptoms (SDI; any symptom) | + any psychotropic medication  residence at a specialised dementia care unit  + male sex | p<0.01  p<0.01  p<0.02 | + age  + care dependency  Institutional guideline on sleep disorders  + proportion of nurses with special knowledge to avoid sleep disturbances  + average of nurses’ years working in elderly care | p=0.42  p=0.53  p=0.50  p=0.54  p=0.33 |
| Zuidema et al 2007^30, 89^ | Clinically significant cases (NPI sleep item; any clinically significant symptom) | + any psychotropic medication  + hypnotics/ sedatives  + antipsychotics  + anxiolytics | p<0.05  p<0.05  p<0.05  p<0.05 | + antidepressants | p>0.05 |
| Zwijsen et al 2014^45^ | Symptoms (NPI sleep item; frequency, severity, mean score) | + staff distress | p<0.05 (severity)  p<0.05 (mean score) | + staff distress | p>0.05 (frequency) |
| *Note: ESS = Epworth Sleepiness Scale; NPI = Neuropsychiatric Inventory; SDI = Sleep Disorders Inventory* | | | | | |
